# Potential roles of the interactions between gut microbiota and metabolites in LPS-induced intrauterine inflammation (IUI) and associated preterm birth (PTB)

**DOI:** 10.1186/s12967-023-04603-8

**Published:** 2024-01-02

**Authors:** Bei Jia, Lijun Tang, Huibing Liu, Wenqian Chen, Qian Chen, Mei Zhong, Ailan Yin

**Affiliations:** Department of Obstetrics and Gynecology, Nanfang Hospital, Southern Medical University, No. 1838 Guangzhou Northern Avenue, Guangzhou, 510515 People’s Republic of China

**Keywords:** Preterm birth (PTB), Intrauterine inflammation (IUI), Gut microbiota, Metabolites, Pyruvic acid

## Abstract

**Background:**

Prenatal exposure to intrauterine inflammation (IUI) is a crucial event in preterm birth (PTB) pathophysiology, increasing the incidence of neurodevelopmental disorders. Gut microbiota and metabolite profile alterations have been reported to be involved in PTB pathophysiology.

**Method and results:**

In this study, IUI-exposed PTB mouse model was established and verified by PTB rate and other perinatal adverse reactions; LPS-indued IUI significantly increased the rates of PTB, apoptosis and inflammation in placenta tissue samples. LPS-induced IUI caused no significant differences in species richness and evenness but significantly altered the species abundance distribution. Non-targeted metabolomics analysis indicated that the metabolite profile of the preterm mice was altered, and differential metabolites were associated with signaling pathways including pyruvate metabolism. Furthermore, a significant positive correlation between *Parasutterella excrementihominis* and S4572761 (Nb-*p*-coumaroyltryptamine) and Mreference-1264 (pyruvic acid), respectively, was observed. Lastly, pyruvic acid treatment partially improved LPS-induced IUI phenotypes and decreased PTB rates and decreased the apoptosis and inflammation in placenta tissue samples.

**Conclusion:**

This study revealed an association among gut microbiota dysbiosis, metabolite profile alterations, and LPS-induced IUI and PTB in mice models. Our investigation revealed the possible involvement of gut microbiota in the pathophysiology of LPS-induced IUI and PTB, which might be mediated by metabolites such as pyruvic acid. Future studies should be conducted to verify the findings through larger sample-sized animal studies and clinical investigations.

**Supplementary Information:**

The online version contains supplementary material available at 10.1186/s12967-023-04603-8.

## Introduction

Preterm birth (PTB) is the leading cause of illness and death in children worldwide, which poses a significant public health concern. Annually, roughly 15 million PTBs occur, resulting in one million fatalities [1]. Even though the mortality rate for preterm babies has improved considerably during the last few decades [2], these newborns remain at an elevated risk of several short- and long-term health inequities, reflecting an early-life genesis of adult illness. Preterm neonates exhibit abnormal pulmonary development, underdeveloped immune systems, and higher oxidative stress, putting them at risk for cognitive/behavioral deficiencies, delayed development, blindness, deafness, gastrointestinal difficulties, and chronic respiratory illnesses [3, 4], and unfavorable metabolic outcomes [5]. Hence, preventing preterm birth has significant consequences for health across the lifetime.

In most instances, spontaneous preterm labor (PTL) is the direct precursor of PTB, whereas intrauterine and systemic infection and inflammation account for 30–40% of this severe clinical state [6, 7]. Prenatal exposure to intrauterine inflammation (IUI) increases the incidence of neurodevelopmental disorders, including cerebral palsy, autism spectrum disorder, cognitive disabilities, and schizophrenia in kids [8, 9]; furthermore, this risk is increased by genetic variations that modify the oxidative stress and inflammatory responses within the body [10, 11]. Localized infection and inflammation were found to be involved in PTB pathophysiology and fetal membranes’ premature rupture. Preterm newborns and their mothers present higher levels of oxidative stress indicators which are adversely related to birth weight and gestational age of newborns [12–15]. Increasing evidence indicates that the inflammation and oxidative stress of mothers and newborns are considered PTB’s etiological factors; however, the underlying mechanisms remain unclear.

The gut microbiota plays a critical role in forming and modifying the immune system and immunological responses. Multiple immunological and metabolic alterations occurring at the placenta during pregnancy aid in preventing rejection of the fetus. Many studies that examined the gut microbiota at longitudinal time points throughout pregnancy demonstrated that the variety and structure of the bacterial community remain mostly consistent [16–18]. Nevertheless, Koren et al. [19] discovered decreased α-diversity and increased β-diversity during early pregnancy compared to late pregnancy, which was shown to be linked to elevated quantity of pro-inflammatory cytokines in feces. Moreover, maternal microbiota transfer from late pregnancy to germ-free animals elicited not only modest inflammation but also increased levels of fecal IL-1β [19]. Dahl et al. analyzed postnatal day 4 fecal specimens from 19 women who delivered preterm and 102 women who delivered at term and discovered lower α-diversity and OTU abundance in the *Bifidobacterium* spp., *Streptococcus* spp., and *Clostridium* spp. families [19]. Several Bifidobacterium strains could suppress in vitro LPS-caused NF-κB activation, IL-8 production, and COX-2 production [20, 21]. Hence, a reduction in bifidobacteria might increase vulnerability into inflammation/infection-caused premature delivery. Shiozaki et al. [22] demonstrated that the OTUs of various *Clostridium difficile* and *Bacillus mimicus* were lower in 10 preterm women compared to 10 full-term women. While these findings demonstrate that intestinal microbial inflammation might be involved in PTB risk modulation, further investigation is required [23, 24].

In this study, IUI-exposed PTB models were established in timed-pregnant CD-1 mice and perinatal adverse reactions were evaluated. Feces samples were collected, intestinal microbial characteristics of model mice were analyzed by 16S RNA gene sequencing, and host circulating metabolites were analyzed using non-targeted metabolomics analysis. Association between gut microbial species and circulating metabolites was analyzed to identify potential metabolites mediating the interactions between gut microbiota and PTB in mice. Finally, the effects of metabolite interference on intrauterine inflammatory phenotypes and perinatal adverse reactions were evaluated.

## Materials and methods

### Mouse resource and regulation

The present study utilized timed-pregnant CD-1® mice purchased from SLAC laboratory animal company (Changsha, China). All procedures involving animal care and treatment were approved by the Animal Care and Use Committee of Nanfang Hospital, Southern Medical University and were performed according to institutional guidelines.

### IUI-exposed PTB models in mice

CD-1 timed pregnant mice were subjected to dissection under isoflurane anesthesia on day 17 of gestation (E17; normal gestation period is 19–20 days). The lowermost two gestational sacs visible in the right lower uterus were exposed, and 100 μL PBS (the control group) or 100 μL PBS added with 25 μg LPS (the modeling group; Escherichia coli O55:B5, Sigma-Aldrich, St. Louis, MO, USA) was injected into the uterine cavity between the first and second gestational sacs of the right uterus. The surgical incision was closed using surgical staples. Birth prior to E19 was considered preterm. After LPS injection, PTB rates were assessed. For the metabolite interference experiment, under anesthesia, LPS-injected mice were given an intraperitoneal injection of 1000 mg/kg of sodium pyruvate or Nb-*p*-counmaroyltryltryptamine (RHAWN, Shanghai, China) at E17; 0.9% sodium chloride was used as the control. Then, similar indexes were monitored.

### Histopathological alterations of placenta tissues

At E18, the placenta tissue samples were collected from mice under anesthesia. The mouse placenta was embedded in paraffin after overnight incubation at 4 °C with 4% PFA. After being deparaffinized, the proximal placenta tissue samples were cut into 4 μm slices, followed by staining with Mayer’s Hematoxylin Solution and 1% Eosin Y Solution as per the manufacturer’s protocol. Following dehydration, cover glasses were mounted using Fisher Scientific’s Permount Mounting Media. Slices were observed and pictures were taken under an optical microscope (Olympus, Kyoto, Japan).

### ELISA detecting inflammatory factor levels in placenta tissues

IL-6, TNF-α, and lipid peroxidation (4-hydroxynonenal) were measured by ELISA using corresponding mouse ELISA kits (Ruixin Biotech, China) according to the manufacturer’s instruction.

### TUNEL detecting apoptosis

The Colorimetric TUNEL Apoptosis Assay Kit from Beyotime (China) was employed to stain the sections according to the manufacturers’ instructions. The brown-stained nucleus indicates apoptosis. Sections were photographed under 400× magnification.

### 16S rRNA gene sequencing

The fecal samples were collected at E18 (control = 7, LPS = 14). Following the manufacturer’s procedure, DNA was extracted from fecal samples with the QIAamp Fast DNA Stool Mini Kit (Qiagen, Germany). The 16S V3–V4 region was amplified using the universal primers previously described [25]. Illumina NovaSeq PE250 was applied to conduct 16S amplicon sequencing. After acquiring the offline raw data (Raw Data), procedures including de-junctioning, filtering, deduplication, base correction and chimeric sequence removal were performed on Raw Data to provide valid sequences for future analysis (Clean Data).

### Bioinformatics and statistical analysis

Herein, Qiime 2’s DADA2 is utilized for denoising Raw Data, which refers to 100% similarity-based clustering, removal and correction of low-quality sequences, algorithm for de-chimerization identification; the de-noised sequences are de-redundant and then Feature (feature, collectively referred to as OTU, ASV) information is obtained. Species annotations are done for the respective sequence of each OTU to obtain the corresponding species information and the information about the distribution of species-based abundance (for example, OTU_table). Then, based on OTU_table, we carried out abundance, α diversity calculations, Venn diagrams, etc. to collect information about species richness and evenness in specimens, common or unique OTUs across multiple specimens and subgroups, etc. Then, further analysis of community structure differences across multiple specimens or subgroups was performed, and the results were represented by descending plots including PCoA and PCA and sample clustering trees. To further investigate the differences within community structure among grouped specimens, using statistical analysis methods including Adonis and Anosim, we tested the comparisons among grouped specimens’ species composition and community structure, analyzed the differences of the α diversity between groups of interest, screened for bacteria differences based on wald test, and finally used PICRUSt2 to predict functions.

Using web analysis tools, we conducted Venn/flower plots and picrust2 functional predictions (https://github.com/picrust/picrust2). Firstly, R software (VennDiagram package) was applied to generate Venn diagram, the list of OTUs held by specimens/groups. Next, jvenn web page for shared and unique OTUs among specimens/groups was employed to visualize the Venn diagram (http://www.bioinformatics.com.cn/static/others/jvenn/example.html).

### Fecal metabolic profiling and data analysis

Fecal samples were placed in a 1.5-mL Eppendorf tube. Then, 20 μL of each of the internal standards Lyso PC17:0 (0.01 mg/mL) and l-2-chlorophenylalanine solution were added. The supernatants from each tube were ultrasonically and centrifugally separated, and then harvested with crystal syringes. After being filtered with 0.22 μm microfilters, the supernatants were transferred to a glass vial for liquid chromatography/mass spectrometry (LC/MS). Next, an AB SCIEX Triple TOF 6600 System and an ACQUITY UHPLC system were applied to analyze the supernatants, as previously reported [26].

### Principal component analysis (PCA) and orthogonal projections to latent structures discriminant analysis (OPLS-DA)

The progenesis QI software (Waters Corporation, Milford, USA) was employed to analyze the obtained raw date. Data processing parameters were applied as follows: precusor tolerance set to 5 ppm, fragment tolerance set to 10 ppm, and product ion threshold set to 5%. The public databases, including the Human Metabolome Database (HMDB), Lipidmaps (v2.3), and METLIN were utilized to identify metabolites. The metabolic changes across experimental groups were visualized using PCA and OPLS-DA models. By conducting a permutation test (200 times), we validated the model and minimized the risk of over-fitting. With the help of PermutMatrix73, we carried out PCA upon Pearson distances. Metabolite differences were screened based on the variable importance in projection (VIP), significance values (P values), and fold-change (FC) values. Generally, metabolites with VIP > 1 were considered to contribute significantly in discrimination interpretation, a FC threshold (FC value ≥ 1.2 or ≤ 0.83) was considered as the cutoff for up/downregulation within the concentration, and a *P*-value of less than 0.05 was regarded as significant.

### Statistical analysis

Each experiment was repeated at least thrice. Data were processed using the GraphPad software (San Diego, CA, USA) and shown as mean ± standard deviation (S.D.). Before processing, all relevant data were examined for normal distribution and variance homogeneity. The Shapiro–Wilk test was utilized for data distribution analysis and the selection of a parametric or non-parametric statistical technique. The Brown–Forsythe test was applied for the comparison of group variances. Kruskal–Wallis was utilized for non-parametric statistical analysis. A Student t-test was used to compare the differences between the two groups. If the data did not have equal variances and had a normal distribution, one-way ANOVA with Dunnett T3 was used to examine differences between two or more groups. If the variances of the data were equivalent, a one-way ANOVA with a Tukey post-hoc test was utilized. The correlation between intestinal microbiota and metabolites was analyzed by Pearson’s Coefficient Analysis. A P value of < 0.05 was considered statistically significant.

## Results

### IUI-related PTB models in mice

**Fig. 1:**
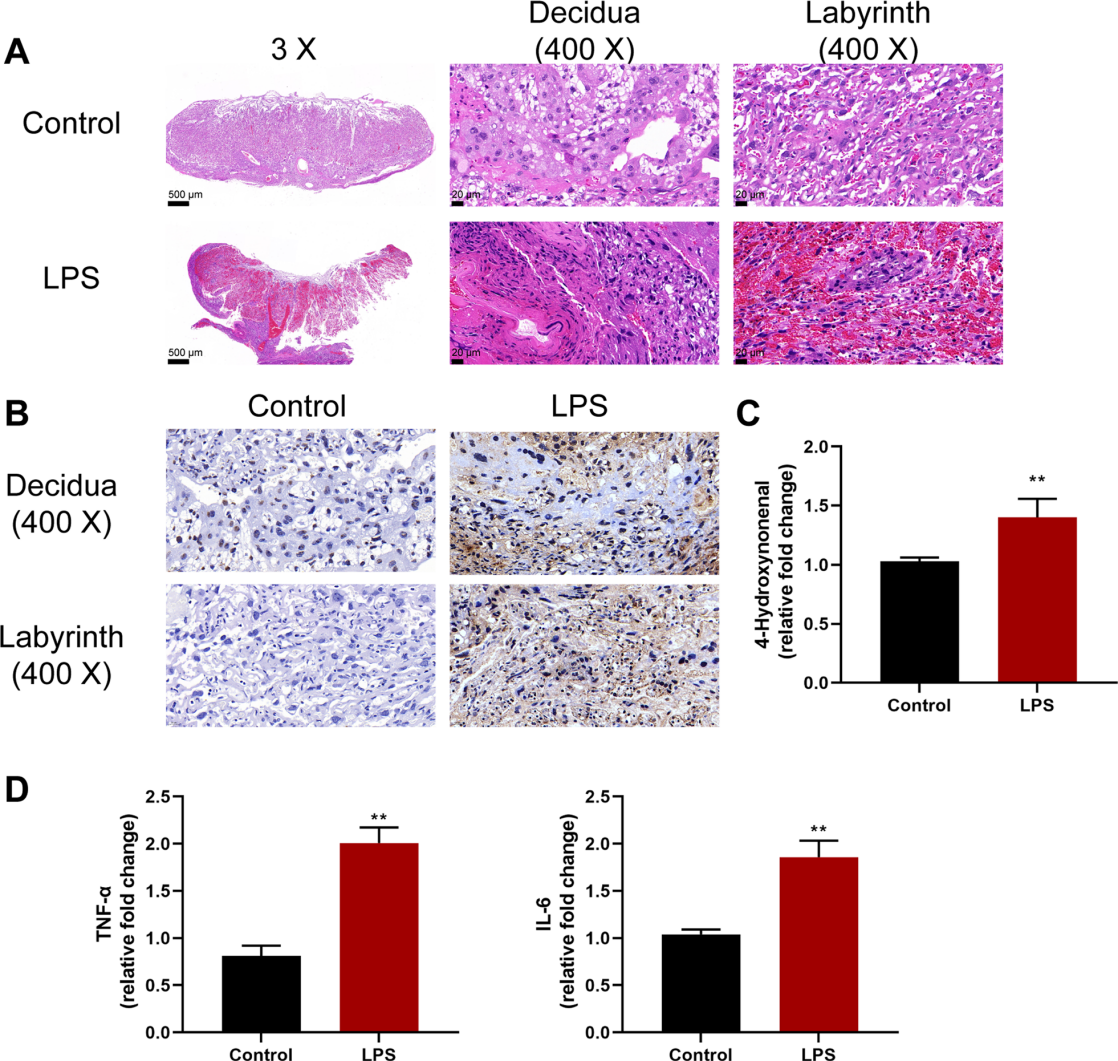
Intrauterine inflammatory (IUI) preterm birth (PTB) model in mice. Animal model was established as described in the M&M section. Control = 7, LPS = 14. **A** Histopathological characteristics of mice placenta tissues were examined using H&E staining; decidua and labyrinth zones are shown. **B** Apoptosis in the decidua and labyrinth zones of mouse placenta tissues was examined using TUNEL staining. **C** Placental oxidative stress marker 4-hydroxynonenal levels in mice placenta tissues were examined using ELISA. **D** The levels of TNF-α and IL-6 in mice placenta tissues were examined using ELISA. **p < 0.01

Animal models were established as described in the M&M section and perinatal adverse reactions were monitored to validate the model. Table 1 shows that the PTB rate of LPS-induced IUI group was 45.45% and significantly higher than the control group. Histopathological examination of placentas by H&E staining shows that, on the fetal side, LPS-induced IUI mice saw structural alterations that were characterized by increased empty spaces and disordered orientation of the villi (Fig. 1A). TUNEL staining revealed considerably higher apoptosis in LPS-stimulated mice’s placentas than normal control (Fig. 1B), indicating further placental disorders upon LPS treatment. Furthermore, 4-hydroxynonenal levels of placental oxidative stress marker were shown to be dramatically increased within LPS-stimulated mice’s placentas than normal control (Fig. 1C). The levels of inflammatory cytokines in placenta tissues, TNF-α and IL-6, were also remarkably increased within LPS-stimulated mice’s placentas than normal control (Fig. 1D). These indexes suggest that LPS-induced IUI-related PTB models were successfully established in mice.

**Table 1:** Preterm rate

	Control	LPS	P-value
Preterm delivery n/N (%)	0/7 (0%)	7/14 (50.00%)	< 0.05

### Gut microbiota disorder in PTB model mice

**Fig. 2:**
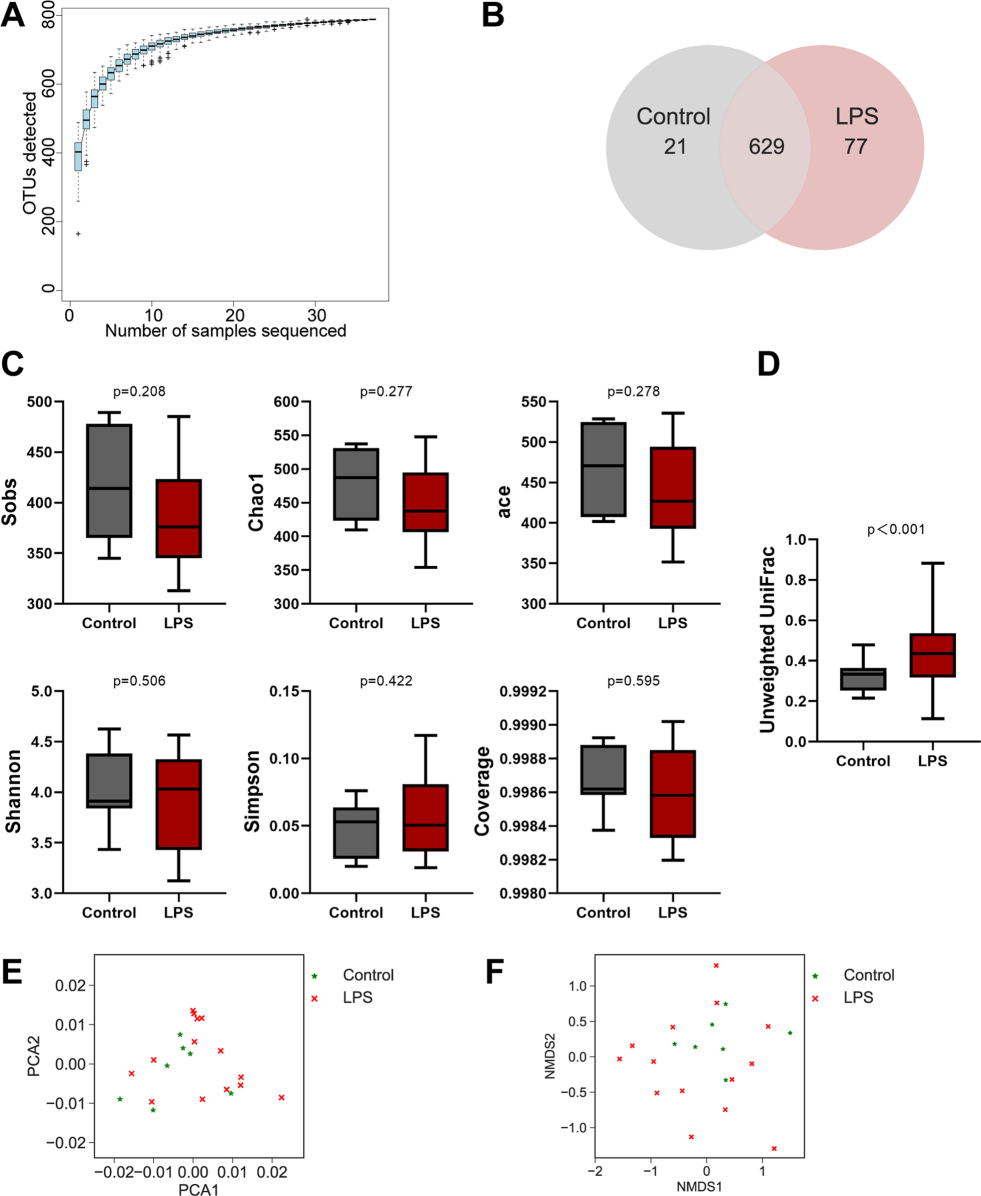
Gut microbiota disorder in PTB model mice. **A** Mice’s intestinal microbiota species accumulation curves. The number of samples sequenced is shown by the horizontal axis, while the number of operational taxonomic units (OTUs) found is represented by the vertical axis. **B** Venn diagram shows changes in the intestinal microbiota of mice in different groups. **C** α-Diversity analysis of intestinal microbiota between the control and PTB modeling (LPS) groups. **D** β-Diversity analysis of intestinal microbiota between the control and PTB modeling (LPS) groups. **E** Principal component analysis (PCA). **F** Nonmetric multidimensional scaling analysis (NMDS)

Concerning the interaction between mice gut microbiota and IUI, feces samples were collected, and the intestinal microbial characteristics of model mice were analyzed by 16S RNA sequencing. As the number of sequences rose, the species accumulation curves became flat, showing that this sequencing depth may represent the entire bacterial species diversity (Fig. 2A). The numbers of the intestinal microbiota of mice were represented using OTUs; the control and LPS groups annotated 650 and 706 OTUs, respectively, and 629 overlapping OTUs were found between the two groups (Fig. 2B). To assess bacterial diversity differences between the LPS group and normal control, we aligned sequences to calculate α-diversity using Sobs, Chao 1, ace, Shannon, Simpson, and coverage index and β-diversity using unweighted UniFrac distance, principal component analysis (PCA), and nonmetric multidimensional scaling analysis (NMDS). α-diversity analysis of intestinal microbiota shows no obvious variations within species richness and evenness between normal control and PTB modeling group (Fig. 2C). However, β-diversity analysis of intestinal microbiota shows that LPS treatment significantly increased the species abundance distribution between two groups (Fig. 2D). Furthermore, PCA and NMDS analyses show that the two groups were not clearly clustered (Fig. 2E, F).

Concerning the annotation of species, the top 10 abundant microbial taxa at the phylum level are *Spirochaetes*, *Firmicutes*, *Deferribacteres*, *Proteobacteria*, *Candidatus_Saccharibacteria*, *Tenericutes*, *Actinobacteria*, *Cyanobacteria*, *Verrucomicrobia*, *Bacteroidetes* (Additional file 1: Fig. S1A, B).

At the genus level, the top 20 abundant microbial taxa are *Anaerotruncus*; *Helicobacter*; *Saccharibacteria*; *Lachnospiracea_incertae_sedis*; *Desulfovibrio*; *Clostridium_XIVa*; *Pseudoflavonifracton*; *Flavonifractor*; *Oscillibacter*; *Clostridium_IV*; *Bacteroides*; *Parabacteroides*; *Alloprevotella*; *Alistipes*; *Lactobacillus*; *Allobaculum*; *Barnesiella*; *Escherichia*; and *Parasutterella* (Additional file 2: Fig. S2A). Additional file 2: Fig. S2B and C show that the abundance of *Parasutterella* was significantly decreased in the LPS-induced IUI group among those microbiota.

**Fig. 3:**
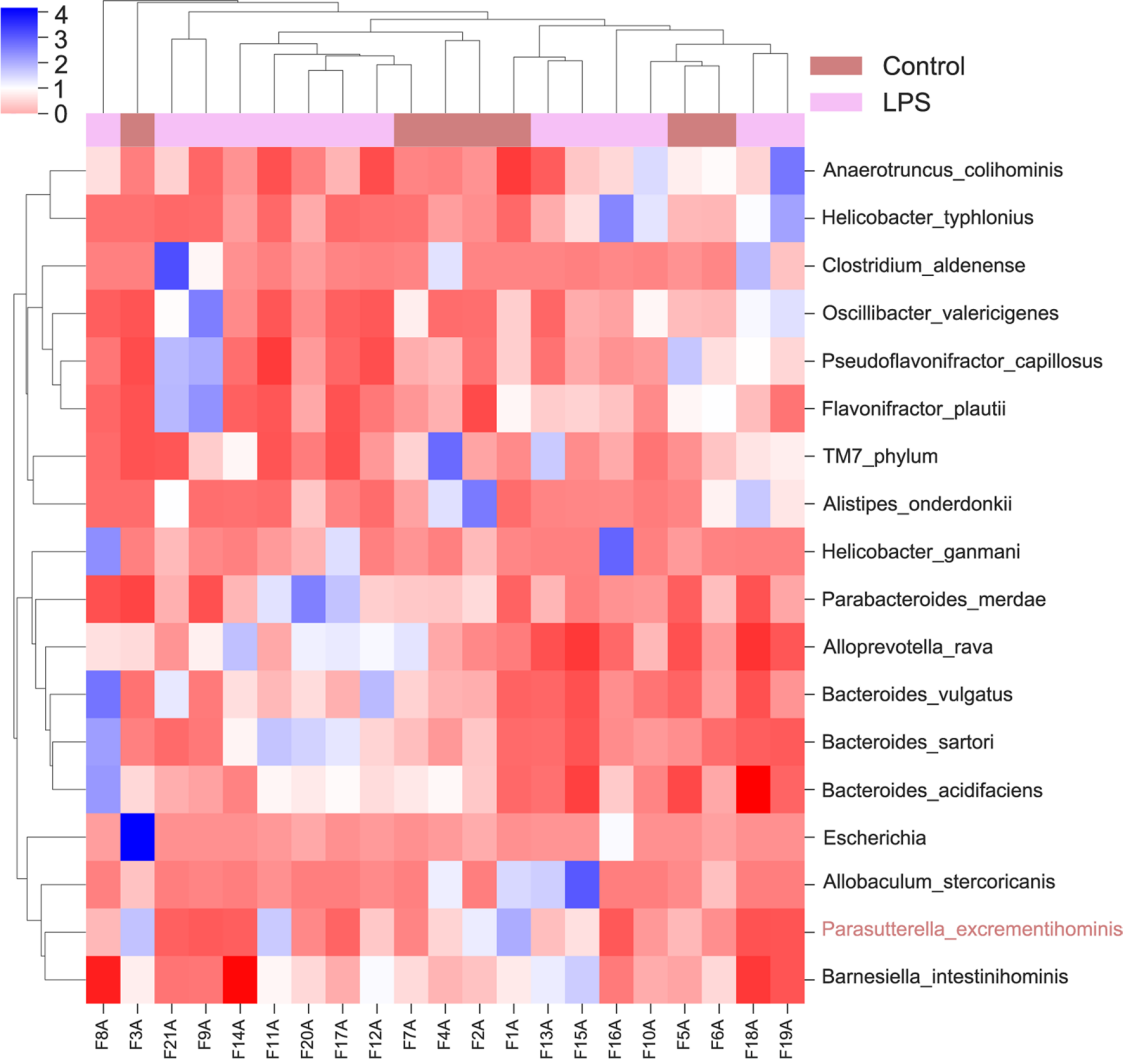
Annotation of intestinal microbiota at the species level. Species of intestinal microbiota in mice from different groups

**Fig. 4:**
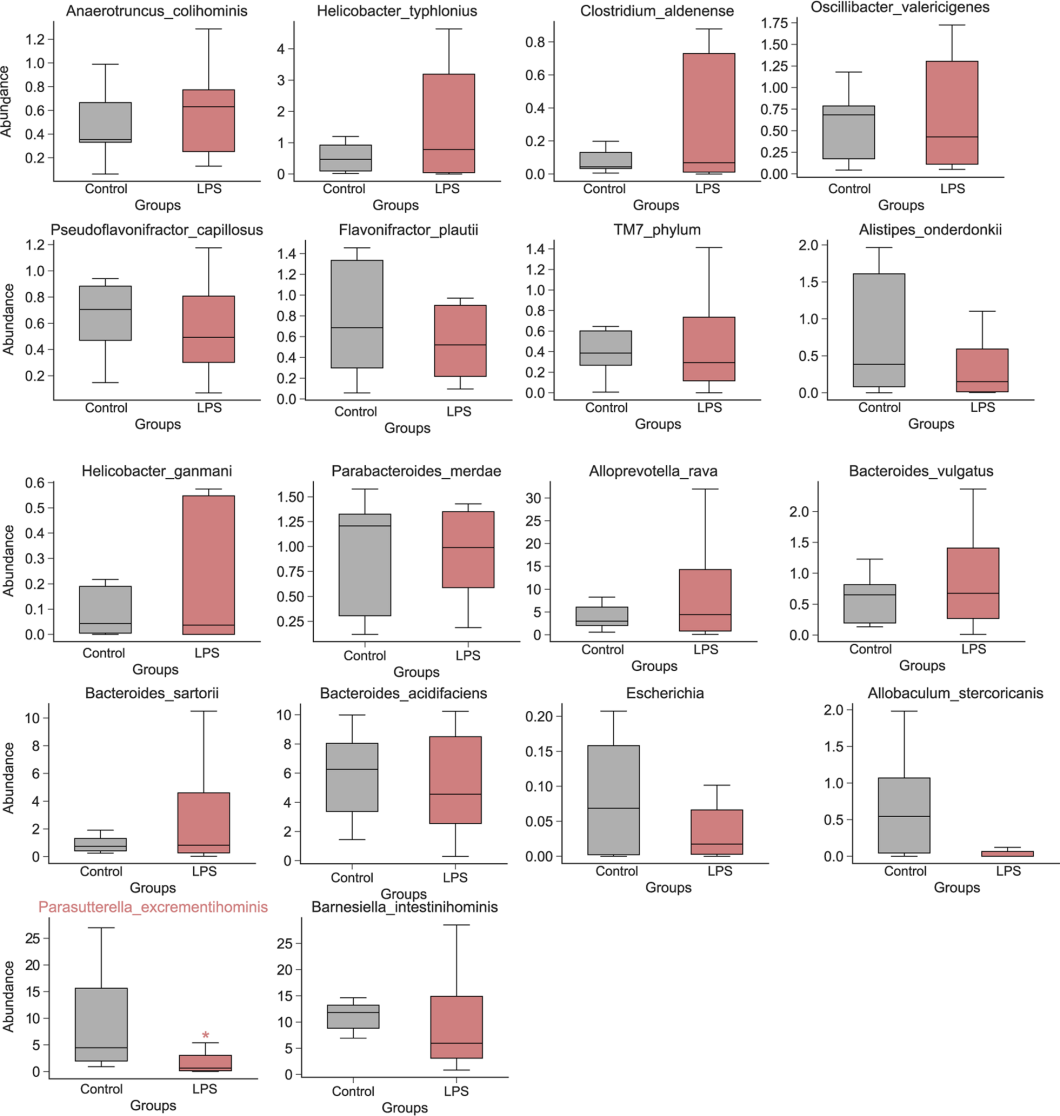
Relative abundance of *Anaerotruncus_colihominis*, *Helicobacter_typhlonius*, *Clostridium_aldenense*, *Oscillibacter_valericigenes*, *Pseudoflavonifractor_capillosus*, *Flavonifractor_plautii*, *TM7_phylum, Alistipes onderdonkii, Helicobacter ganmani*, *Parabacteroides merdae*, *Alloprevotella rava*, *Bacteroides_vulgatus*, *Bacteroides sartori*, *Bacteroides acidifaciens*, *Escherichia*, *Allobaculum_stercoricanis*, *Parasutterella excrementihominis*, and *Barnesiella intestinihominis*. *p < 0.05

At the species level, the top 20 abundant microbial taxa are *Anaerotruncus_colihominis*; *Helicobacter_typhlonius*; *Clostridium_aldenense*; *Oscillibacter_valericigenes*; *Pseudoflavonifractor_capillosus*; *Flavonifractor_plautii*; *TM7_phylum*; *Alistipes onderdonkii*; *Helicobacter ganmani*; *Parabacteroides merdae*; *Alloprevotella rava*; *Bacteroides_vulgatus*; *Bacteroides sartori*; *Bacteroides acidifaciens*; *Escherichia*; *Allobaculum_stercoricanis*; *Parasutterella excrementihominis*; and *Barnesiella intestinihominis* (Fig. 3). *Parasutterella excrementihominis* had the highest relative abundance difference between the two groups (Fig. 4, p < 0.05). The relative abundance of these species in the LPS and control groups is shown in Fig. 4.

### Metabolic differences of mice in different groups

**Fig. 5:**
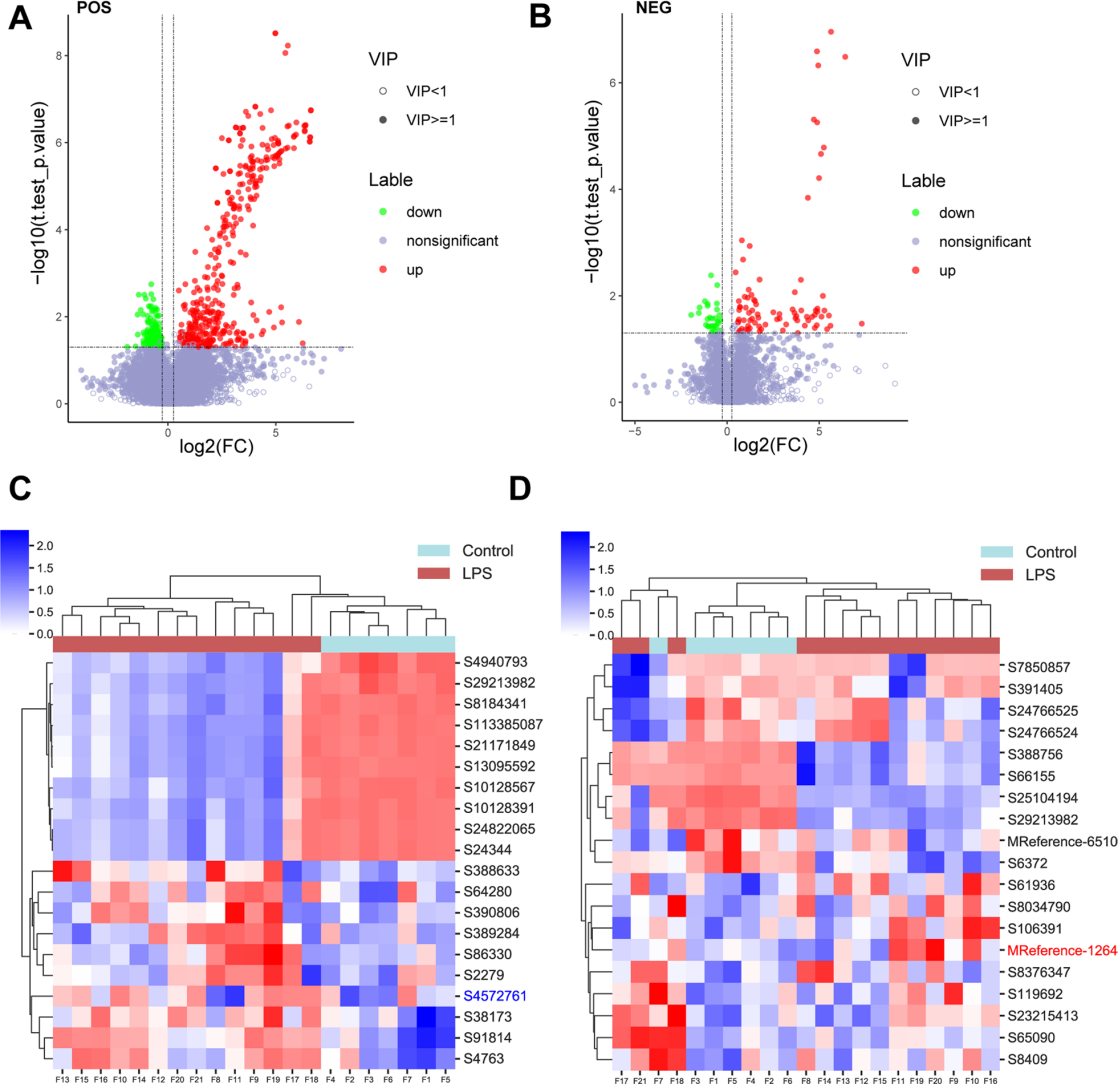
Metabolic differences of mice in different groups. **A**–**D** Volcano plot (**A**, **B**) and hierarchical clustering heatmap (**C**, **D**) show differential accumulation of metabolites in the control and PTB groups. **C** POS Mode: S4940793: Sulukast; S29213982: (3β,4β,5α,15α,16β,25s)-4,5,8,15,16,26-hexahydroxycholestan-3-yl 2-*o*-methyl-β-d-xylopyranoside; S8184341: *N*-acetyldeferoxamine; S113385087: (2β,3β,5β,22r)-2,20,22-trihydroxy-6-oxocholesta-7,14-dien-3-yl β-d-glucopyranoside; S21171849: 1-*o*-[(1,3,24,28-tetrahydroxy-4,14-dimethyl-22,28-epoxy-9,19-cycloergostan-4-yl)carbonyl]hexopyranose; S13095592: Timosaponin aiii; S10128567: Actodigin; S10128391: cyclic-3,20-bis(1,2-ethanediyl acetal)-11α-(acetyloxy)-5α,6α-epoxypregnane-3,20-dione; S24822065: prostaglandin F_2α_ serinol amide; S24344: hydrocortisone butyrate (jp15/usp); S388633: Z-Gly-Pro; S64280: promolate; S390806: cinnamodial; S389284: 3-(4-hydroxy-3,5-diiodophenyl)lactic acid; S86330: 3-bromo-1-thiolane-1,1-dione; S2279: betaxolol; S4572761: (2e)-3-(4-hydroxyphenyl)-*n*-[2-(1h-indol-3-yl)ethyl]acrylamide/Nb-*p*-coumaroyltryptamine; S38173: medroxalol; S91814: (−)-lobeline; S4763: propafenone. **D** NEG mode: S7850857: fenpyroximate; S391405: *N*-acetylleucylleucine; S24766525: 1-[(9z)-hexadecenoyl]-*sn*-glycero-3-phosphocholine; S24766524: lysophosphatidylcholine 14:1(9z)/0:0; S388756: {[(3α,7α,8ξ,10ξ,12α,13ξ,17ξ,20ξ)-3,7,12-trihydroxy-24-oxocholan-24-yl]amino}methyl hydrogen sulfate; S66155: ergocornine; S25104194: mapracorat; S29213982: (3β,4β,5α,15α,16β,25s)-4,5,8,15,16,26-hexahydroxycholestan-3-yl 2-*o*-methyl-β-d-xylopyranoside; MReference-6510: cannabigerolic acid; S6372: chlorfenethol; S61936: arbekacin; S8034790: 3,10-dihydroxydecanoic acid; S106391: 2-amino-2-deoxy-4-*o*-β-d-galactopyranosyl-d-glucose; MReference-1264: pyruvic acid; S8376347: (−)-acutumine; S119692: cystathionine ketamine; S23215413: (9z,12z,15z)-*n*-[2-(3,4-dihydroxyphenyl)ethyl]-9,12,15-octadecatrienamide; S65090: icotidine; S8409: *N*-butyl lactate

Table 2 shows that under positive (POS) mode, we identified 562 metabolites, with 390 increased and 172 decreased within the LPS group; under negative (NEG) mode, we identified 110 metabolites, with 81 increased and 29 decreased within the LPS group. The metabolites of differential accumulation in the control and PTB groups were shown in the Volcano plot [positive ion (pos), negative ion (neg); Fig. 5A, B]. The hierarchical clustering heatmap shows significantly up-regulated and down-regulated metabolites with Top 10 VIP scores (p.value < 0.05, Fig. 5C, D, Table 3). Under POS mode, Sulukast, (3β,4β,5α,15α,16β,25s)-4,5,8,15,16,26-hexahydroxycholestan-3-yl 2-*o*-methyl-β-d-xylopyranoside, *N*-acetyldeferoxamine, (2β,3β,5β,22r)-2,20,22-trihydroxy-6-oxocholesta-7,14-dien-3-yl β-d-glucopyranoside, 1-*o*-[(1,3,24,28-tetrahydroxy-4,14-dimethyl-22,28-epoxy-9,19-cycloergostan-4-yl)carbonyl]hexopyranose, Timosaponin aiii, Actodigin, Cyclic-3,20-bis(1,2-ethanediyl acetal)-11α-(acetyloxy)-5α,6α-epoxypregnane-3,20-dione, Prostaglandin F_2α_ serinol amide, Hydrocortisone butyrate (jp15/usp), Z-Gly-Pro, Promolate, Cinnamodial, 3-(4-hydroxy-3,5-diiodophenyl)lactic acid, 3-bromo-1-thiolane-1,1-dione, Betaxolol, (2e)-3-(4-hydroxyphenyl)-*n*-[2-(1h-indol-3-yl)ethyl]acrylamide/Nb-*p*-coumaroyltryptamine, Medroxalol, (−)-lobeline, and Propafenone were the top 20 metabolites with the highest VIP scores. Under NEG mode, Fenpyroximate, *N*-acetylleucylleucine, 1-[(9z)-hexadecenoyl]-*sn*-glycero-3-phosphocholine, lysophosphatidylcholine 14:1(9z)/0:0, {[(3α,7α,8ξ,10ξ,12α,13ξ,17ξ,20ξ)-3,7,12-trihydroxy-24-oxocholan-24-yl]amino}methyl hydrogen sulfate, Ergocornine, Mapracorat, (3β,4β,5α,15α,16β,25s)-4,5,8,15,16,26-hexahydroxycholestan-3-yl 2-*o*-methyl-β-d-xylopyranoside, Cannabigerolic acid, Chlorfenethol, Chlorfenethol, Arbekacin, 3,10-dihydroxydecanoic acid, 2-amino-2-deoxy-4-*o*β-d-galactopyranosyl-d-glucose, Pyruvic acid, (−)-acutumine, Cystathionine ketimine, (9z,12z,15z)-*n*-[2-(3,4-dihydroxyphenyl)ethyl]-9,12,15-octadecatrienamide, Icotidine, and *N*-butyl lactate were the top 20 metabolites with the highest VIP scores.

**Table 2:** Statistics of differential metabolites

Mode	Group	Total number of differential metabolites	Up	Down
Pos	LPS vs control	562	390	172
Neg	LPS vs control	110	81	29

**Table 3:** The up-regulated and down-regulated metabolites with top 10 VIP scores in positive mode and negative mode

	ID	Name	Compound ID	Formula	Molecular weight
POS	S4940793	Sulukast	4.817_472.2524	C25 H36 N4 O3 S	472.2524
	S29213982	(3β,4β,5α,15α,16β,25s)-4,5,8,15,16,26-Hexahydroxycholestan-3-yl 2-*o*-methyl-β-d-xylopyranoside	9.354_630.3984	C33 H58 O11	630.3984
	S8184341	*N*-Acetyldeferoxamine	8.127_602.3668	C27 H50 N6 O9	602.3668
	S113385087	(2β,3β,5β,22r)-2,20,22-Trihydroxy-6-oxocholesta-7,14-dien-3-yl β-d-glucopyranoside	9.382_608.3536	C33 H52 O10	608.3536
	S21171849	1-*o*-[(1,3,24,28-Tetrahydroxy-4,14-dimethyl-22,28-epoxy-9,19-cycloergostan-4-yl)carbonyl]hexopyranose	9.352_696.406	C37 H60 O12	696.406
	S13095592	Timosaponin aiii	9.343_740.4327	C39 H64 O13	740.4327
	S10128567	Actodigin	8.093_536.2972	C29 H44 O9	536.2972
	S10128391	Cyclic-3,20-bis(1,2-ethanediyl acetal)-11α-(acetyloxy)-5α,6α-epoxypregnane-3,20-dione	9.385_476.2756	C27 H40 O7	476.2756
	S24822065	Prostaglandin f2α serinol amide	9.398_427.2936	C23 H41 N O6	427.2936
	S24344	Hydrocortisone butyrate (jp15/usp)	9.397_432.2491	C25 H36 O6	432.2491
	S388633	Z-Gly-Pro	0.681_306.1219	C15 H18 N2 O5	306.1219
	S64280	Promolate	0.692_293.1628	C16 H23 N O4	293.1628
	S390806	Cinnamodial	0.667_308.1627	C17 H24 O5	308.1627
	S389284	3-(4-Hydroxy-3,5-diiodophenyl)lactic acid	0.618_433.8493	C9 H8 I2 O4	433.8493
	S86330	3-Bromo-1-thiolane-1,1-dione	0.597_197.9359	C4 H7 Br O2 S	197.9359
	S2279	Betaxolol	0.595_307.2151	C18 H29 N O3	307.2151
	S4572761	(2e)-3-(4-Hydroxyphenyl)-*n*-[2-(1h-indol-3-yl)ethyl]acrylamide/Nb-p-coumaroyltryptamine	6.415_306.1368	C19 H18 N2 O2	306.1368
	S38173	Medroxalol	0.727_372.1701	C20 H24 N2 O5	372.1701
	S91814	(−)-Lobeline	0.741_337.2051	C22 H27 N O2	337.2051
	S4763	Propafenone	0.738_341.1999	C21 H27 N O3	341.1999
NEG	S7850857	Fenpyroximate	7.486_421.1994	C24 H27 N3 O4	421.1994
	S391405	*N*-Acetylleucylleucine	5.644_286.1886	C14 H26 N2 O4	286.1886
	S24766525	1-[(9z)-Hexadecenoyl]-*sn*-glycero-3-phosphocholine	9.585_493.3155	C24 H48 N O7 P	493.3155
	S24766524	Lysophosphatidylcholine 14:1(9z)/0:0	9.214_465.2846	C22 H44 N O7 P	465.2846
	S388756	{[(3α,7α,8ξ,10ξ,12α,13ξ,17ξ,20ξ)-3,7,12-Trihydroxy-24-oxocholan-24-yl]amino}methyl hydrogen sulfate	8.751_517.2697	C25 H43 N O8 S	517.2697
	S66155	Ergocornine	8.793_561.2966	C31 H39 N5 O5	561.2966
	S25104194	Mapracorat	6.158_462.1915	C25 H26 F4 N2 O2	462.1915
	S29213982	(3β,4β,5α,15α,16β,25s)-4,5,8,15,16,26-Hexahydroxycholestan-3-yl 2-*o*-methyl-β-d-xylopyranoside	8.983_630.398	C33 H58 O11	630.398
	MReference-6510	Cannabigerolic acid	8.249_360.2294	C22 H32 O4	360.2294
	S6372	Chlorfenethol	0.828_266.0258	C14 H12 Cl2 O	266.0258
	S61936	Arbekacin	7.692_552.3104	C22 H44 N6 O10	552.3104
	S8034790	3,10-Dihydroxydecanoic acid	4.18_204.136	C10 H20 O4	204.136
	S106391	2-Amino-2-deoxy-4-o-β-d-galactopyranosyl-d-glucose	0.709_341.1316	C12 H23 N O10	341.1316
	MReference-1264	Pyruvic acid	0.634_88.0161	C3 H4 O3	88.0161
	S8376347	(−)-Acutumine	4.973_397.1302	C19 H24 Cl N O6	397.1302
	S119692	Cystathionine ketimine	2.777_203.025	C7 H9 N O4 S	203.025
	S23215413	(9z,12z,15z)-*n*-[2-(3,4-Dihydroxyphenyl)ethyl]-9,12,15-octadecatrienamide	9.46_413.294	C26 H39 N O3	413.294
	S65090	Icotidine	4.813_379.2023	C21 H25 N5 O2	379.2023
	S8409	*N*-Butyl lactate	3.677_146.0942	C7 H14 O3	146.0942

### Signaling pathways associated with differential metabolites under POS and NEG data acquisition models

**Fig. 6:**
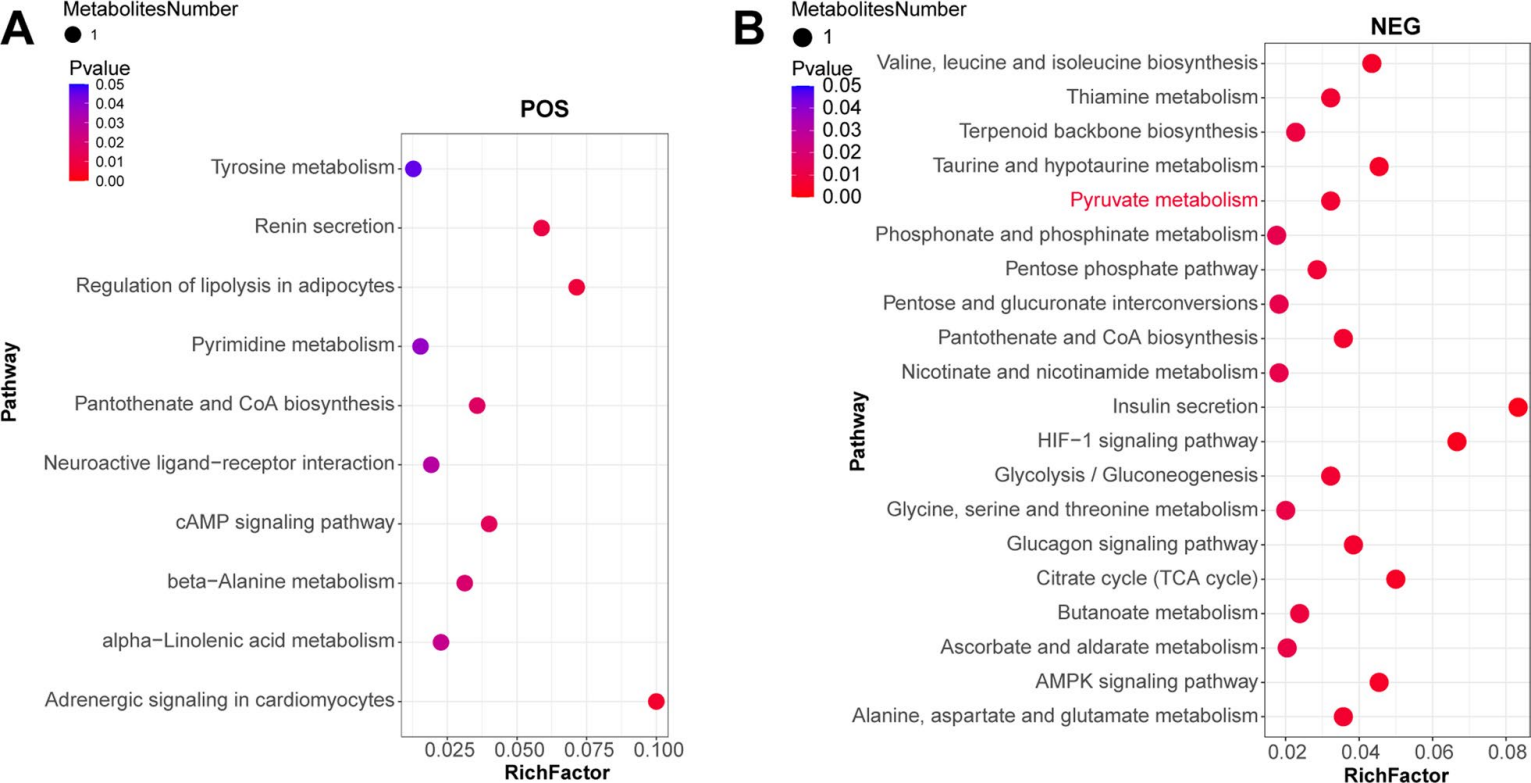
Signaling pathways associated with differential metabolites under POS (**A**) and NEG (**B**) data acquisition models

Positive ion-captured metabolites are mainly associated with cardiomyocyte adrenergic signaling, lipolytic regulation, renin secretion, cAMP signaling pathway, pantothenic acid and coenzyme A biosynthesis, β-Alanine metabolism, linolenic acid metabolism, stimulation of interactions in neural tissue, pyrimidine metabolism, tyrosine metabolism, and other biological functions (Fig. 6A).

Negative ion-captured metabolites are mainly related to insulin secretion, HIF-1 signaling pathway, TCA cycle, taurine metabolism, AMPK signaling pathway, anabolic metabolism of various amino acids such as valine/leucine/isoleucine/aspartate/glutamate, glucagon signaling pathway, pantothenic acid and coenzyme A biosynthesis, thiamine metabolism, glycolysis, pyruvate metabolism, carbon metabolism, and other biological functions-related (Fig. 6B).

### Association between gut microbial species and circulating metabolites

**Fig. 7:**
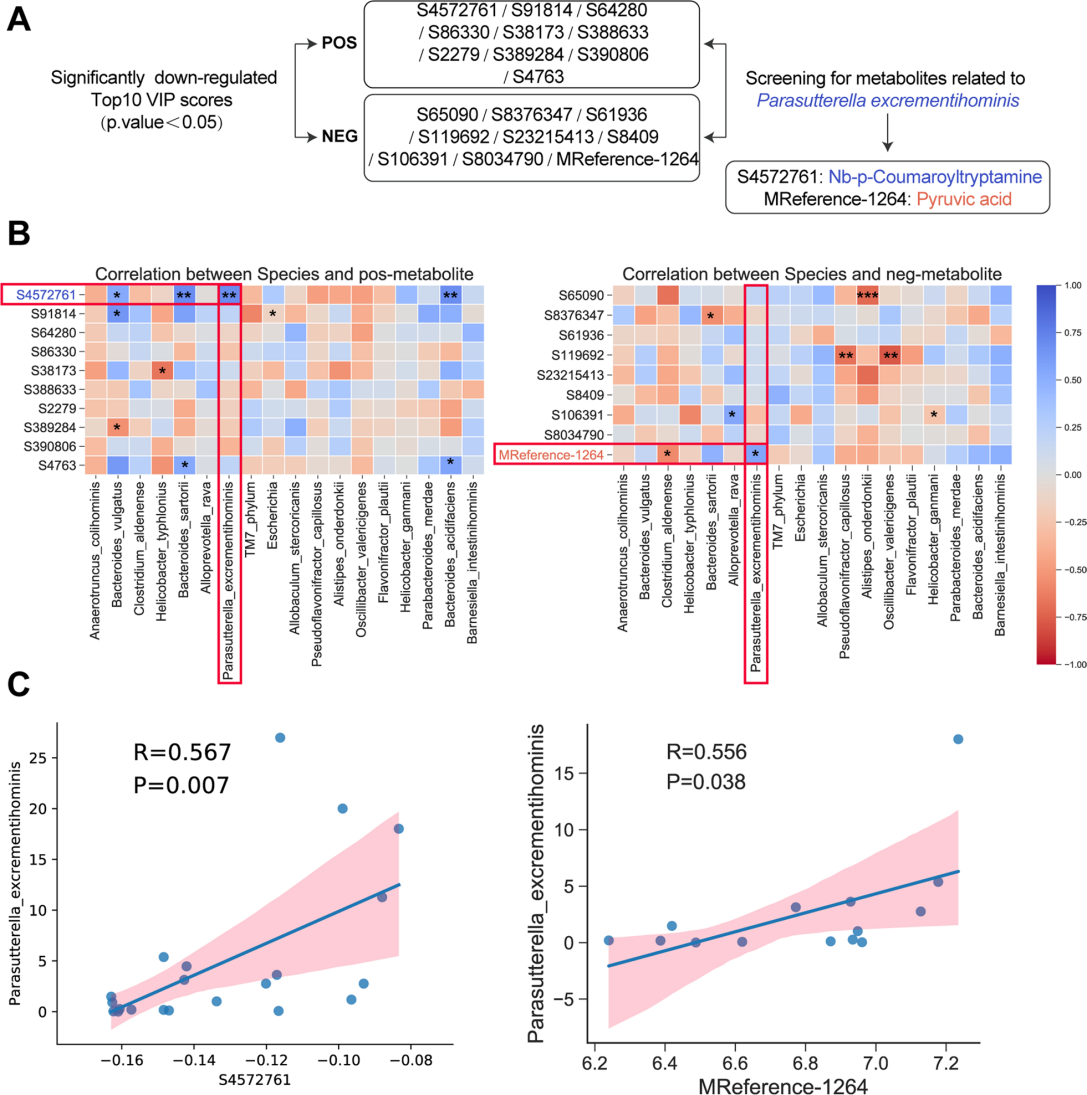
Association between gut microbial species and circulating metabolites. **A** Bioinformatics process for screening metabolites associated with the intestinal microbiota. **B** Differential intestinal microbiota (species) related metabolites. **C** Correlation between *Parasutterella_excrementithomins* and s4572761 (Nb-*p*-coumaroyltryptamine) and MReference-1264 (pyruvic acid), respectively

Considering the disordered gut microbiota and metabolites, the potential association between gut microbiota species and metabolites was analyzed. The downregulated metabolites in the LPS group with the top 10 VIP score metabolites in positive and negative ion modes were selected. Then, the metabolites correlated with the abundance of *Parasutterella excrementihominis* were further selected. Among the two metabolites, S4572761 (Nb-*p*-coumaroyltryptamine) [27] and Mreference-1264 (pryuvic acid) [28], has been reported could inhibit inflammation. Figure 7B shows the association between differential gut microbiota (species) and positive/negative ion-captured metabolites. As shown in Fig. 7C, a significant positive correlation between *Parasutterella excrementihominis* and S4572761 (Nb-*p*-coumaroyltryptamine)/mreference-1264(pyruvic acid) was observed.

### Metabolite interference alleviates intrauterine inflammatory phenotypes affecting perinatal adverse reactions

**Fig. 8:**
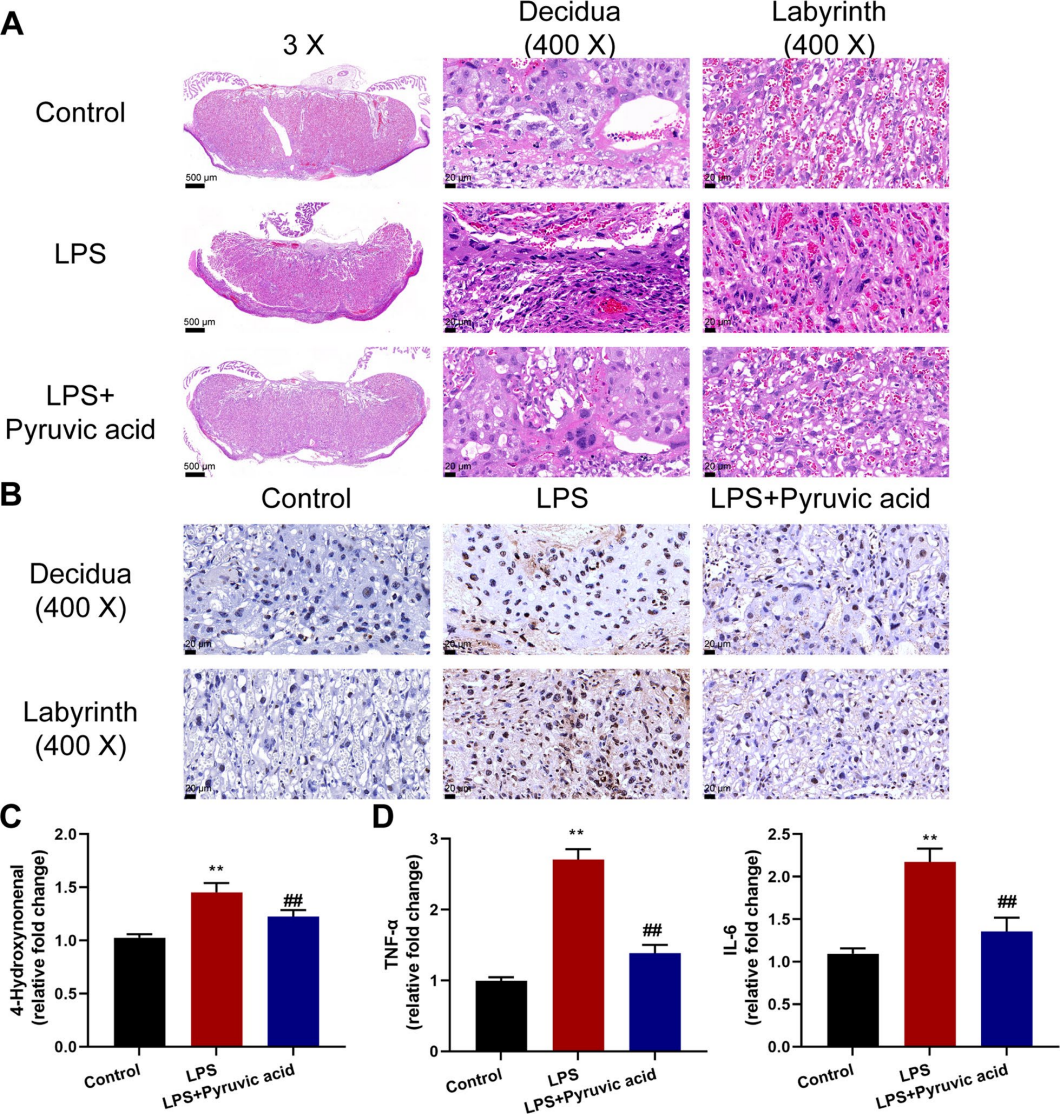
Metabolite interference alleviates intrauterine inflammatory phenotypes affecting perinatal adverse reactions. Animal models were established and pyruvic acid treatment was administrated as described in the M&M section. **A** Histopathological characteristics of mice placenta tissues were examined using H&E staining; decidua and labyrinth zones are shown. **B** Apoptosis in the decidua and labyrinth zones of mouse placenta tissues was examined using TUNEL stainin€. **C** Placental oxidative stress marker 4-hydroxynonenal levels in mice placenta tissues were examined using ELISA. **D** The levels of TNF-α and IL-6 in mice placenta tissues were examined using ELISA. Control = 6, LPS = 15, LPS+ pyruvic acid = 15. **p < 0.01 vs. control group; ^##^p < 0.01 vs. LPS group

Since *Parasutterella excrementihominis* was significantly positively correlated with Nb-*p*-coumaroyltryptamine and pyruvic acid, next, LPS-induced IUI mice models were established, and Nb-*p*-coumaroyltryptamine or pyruvic acid treatment was administrated to investigate the specific effects of Nb-*p*-coumaroyltryptamine or pyruvic acid on intrauterine inflammatory phenotypes affecting perinatal adverse reactions. Table 4 shows that pyruvic acid treatment significantly decreased the PTB rate from 53.3 to 20% compared with the IUI model group (p = 0.028). Histopathological examination of the placentas by H&E staining shows that LPS-induced structural alterations partially improved by pyruvic acid treatment (Fig. 8A). Consistently, TUNEL staining revealed that pyruvic acid treatment suppressed apoptosis in the placentas of LPS-treated mice (Fig. 8B). Also, the pyruvic acid treatment decreased the placental oxidative stress marker 4-hydroxynonenal levels (Fig. 8C) and the levels of placental inflammation and apoptosis markers, TNF-α and IL-6, compared with the LPS-induced IUI group (Fig. 8D). Therefore, pyruvic acid treatment partially improved LPS-induced IUI phenotypes and perinatal adverse reactions in PTB mice. Importantly, Nb-*p*-coumaroyltryptamine also partially improved LPS-induced histopathological alterations (Additional file 3: Fig. S3A), apoptosis in the placentas (Additional file 3: Fig. S3B), the placental oxidative stress marker 4-hydroxynonenal levels (Additional file 3: Fig. S3C), and the levels of placental inflammation and apoptosis markers, TNF-α and IL-6 (Additional file 3: Fig. S3D); however, the improvement of preterm rate was not as effective as pyruvic acid treatment (Additional file 4: Table S1, the PTB rate reduced 14.29%, p = 0.040).

## Discussion

In this study, IUI-exposed PTB models were established and verified by PTB rate and other perinatal adverse reactions; LPS-indued IUI significantly increased the rates of PTB. LPS-induced IUI caused no significant differences in species richness and evenness but significantly altered the species abundance distribution. Non-targeted metabolomics analysis indicated that the metabolite profile of the preterm mice was altered, and differential metabolites were associated with signaling pathways including pyruvate metabolism. Furthermore, a significant positive correlation between *Parasutterella excrementihominis* and Mreference-1264 (pyruvic acid) and S4572761 (Nb-*p*-coumaroyltryptamine) was observed. Lastly, pyruvic acid treatment partially improved LPS-induced IUI phenotypes and decreased rates of PTB. Although Nb-*p*-coumaroyltryptamine also partially improved LPS-induced IUI phenotypes, the improvement of PTB rate by Nb-*p*-coumaroyltryptamine was not as effective as pyruvic acid treatment.

**Table 4:** Preterm rate after metabolites (pyruvic acid) intervention

	Control	LPS	LPS + pyruvic acid	P-value
Preterm delivery n/N (%)	0/6 (0%)	8/15 (53.33%)	3/15 (20.00%)	0.028

In LPS-indued IUI models, significantly increased rates of PTB and increased apoptosis in the placenta were observed, suggesting that LPS-induced IUI might be the crucial pre-event of PTB. In addition, significantly increased TNF-α and IL-6 levels were observed in model mice placenta tissues. Chorioamnionitis, characterized as neutrophilic infiltration of the placenta, fetal membranes, and amniotic fluid, is the histologic correlate of IUI [29, 30]. Neutrophils are the predominant leukocyte during IUI and are stimulated to release pro-inflammatory mediators [31, 32]. IL-6 and PGs are confirmed inflammatory indicators that mediate premature labor [33, 34]. TNF-blockade lowered poor pregnancy outcomes in animal models of IUI including neutrophil infiltration into the placenta [35, 36]. Furthermore, after intrauterine LPS administration, we detected elevated levels of the oxidative stress marker 4-hydroxynonenal in the placentas of model mice. Oxidative stress is the key factor in the etiology of PTB. Previous research indicates that cord blood from preterm neonates has much higher levels of oxidative stress indicators than cord blood from term newborns [12, 14]. Treatment with antioxidants (curcumin, naringenin, or apigenin) decreased the production of LPS-induced IL-6, IL-8, and COX-2 in human prenatal tissues (placenta, fetal membranes, and myometrium) [37]. Many antioxidants have shown protective effects on PTB, fetal mortality, and intrauterine growth restriction in animal models [38, 39]. Hence, oxidative stress correlates highly with PTB and other unfavorable birth outcomes.

A host’s gut microbiota is critical in forming and modifying immune responses [40]. The dysbiosis of gut microorganisms is a risk factor for the development of inflammation [41, 42]. Reduced gut microbial diversity is associated with an increased risk of gastrointestinal disorders and proinflammatory characteristics [43, 44], and reduced richness is a frequent marker of chronic disease [45]. During T1 of the miscarriage group, Liu et al. [46] discovered a reduced gut microbiota and an increased ratio of *Firmicutes* to *Bacteroidetes*. Under prenatal stress, the disturbed *Parasutterella excrementihominis* seems crucial to inflammatory and metabolic abnormalities. Likewise, Ju et al. [47] have revealed a vital function for *Parasutterella* in gut health by revealing that this genus is not only a recognized colonizer of the mouse gut but also a succinate generator. Previous research has shown that succinate indirectly promotes the formation of fermenters such as *Clostridiales*, therefore protecting newborn mice against infection [48]. In our work, the abundance of *Parasutterella excrementihomini* was considerably reduced in the LPS-induced IUI group, demonstrating that, most possibly, the proinflammatory effects of the microbiome in PTB mice are related to widespread dysbiosis rather than a specific pathogen.

The fact that a high fraction of blood metabolites is derived from the gut suggests that metabolites play a major role in microbiota-cytokine interactions [49, 50]. Liu et al. [46] also demonstrated that microbial metabolic activities have a significant effect on cytokine production. Thus, metabolites were analyzed using non-targeted metabolomics analysis. In our study, the metabolite profile of the preterm mice was altered, and differential metabolites were associated with signaling pathways including pyruvate metabolism. Among the top 20 metabolites with the highest VIP scores under POS and NEG modes, respectively, betaxolol has been reported to efficiently treat the arterial hypertension in pregnancy [51, 52]. Another metabolite, Arbekacin, is classified as an aminoglycoside antibiotic that exhibits stability against the majority of aminoglycoside-inactivating enzymes. It has potent bactericidal properties against a diverse range of Gram-negative bacilli and Gram-positive cocci [53, 54]. Pyruvic acid is one of the principal sources of acetyl-CoA and an essential chemical for energy delivery to living cells through the TCA cycle. Pyruvate may be produced from glucose by glycolysis, gluconeogenesis, or glutaminolysis, in which glutamine is converted to glutamate and pyruvic acid, among other products. In addition, greater glutamine levels and glutamine/glutamate ratios may be associated with elevated glycoprotein P1 levels, which may indicate renal disease or fetal development limitation [55]. Pyruvic acid in the amniotic fluid has been regarded as one of the metabolic signatures associated with preterm delivery [56]. In this study, a significant positive correlation between *Parasutterella excrementihominis* and Mreference-1264 (Pyruvic acid) was observed; therefore, the specific functions of pyruvic acid treatment on LPS-induced IUI were investigated. As expected, pyruvic acid treatment improved LPS-induced IUI phenotypes and perinatal adverse reactions in PTB mice. Interestingly, another metabolite Nb-*p*-coumaroyltryptamine has been reported to inhibit the activation of the JNK/c-Jun signaling pathway in RAW264.7 cells stimulated by LPS [27]. In this study, although Nb-*p*-coumaroyltryptamine also partially improved LPS-induced IUI phenotypes, the improvement of PTB rate by Nb-*p*-coumaroyltryptamine was not as effective as pyruvic acid treatment. Therefore, pyruvic acid might mediate the interaction between gut microbiota and LPS-induced IUI and PTB.

In conclusion, this study revealed an association among gut microbiota dysbiosis, metabolite profile alterations, and LPS-induced IUI and PTB in mice models. Our investigation revealed the possible involvement of gut microbiota in the pathophysiology of LPS-induced IUI and PTB, which might be mediated by metabolites such as pyruvic acid. Future studies should be conducted to verify the findings through larger sample-sized animal studies and clinical investigations.

### Supplementary Information

**Additional file 1: Figure S1.** Annotation on intestinal microbiota at the phylum level. (A) Phyla of intestinal microbiota in mice from different groups. (B) Relative abundance of these phyla of intestinal microbiota.

**Additional file 2: Figure S2.** Annotation on intestinal microbiota at the genus level. (A) Genera of intestinal microbiota in mice from different groups. (B, C) Relative abundance of these genera of intestinal microbiota. *p < 0.05.

**Additional file 3: Figure S3.** Effects of Nb-*p*-coumaroyltryptamine (s4572761) on intrauterine inflammatory phenotypes affecting perinatal adverse reactions. Animal models were established and Nb-*p*-coumaroyltryptamine treatment was administrated as described in the M&M section. (A) Histopathological characteristics of mice placenta tissues were examined using H&E staining; decidua and labyrinth zones are shown. (B) Apoptosis in the decidua and labyrinth zones of mouse placenta tissues was examined using TUNEL staining. (C) Placental oxidative stress marker 4-hydroxynonenal levels in mice placenta tissues were examined using ELISA. (D) The levels of TNF-α and IL-6 in mice placenta tissues were examined using ELISA. Control = 7, LPS = 14, LPS+ Nb-*p*-coumaroyltryptamine = 14. **p < 0.01 vs. control group; ^##^p < 0.01 vs. LPS group.

**Additional file 4: Table S1.** Preterm rate after metabolites intervention (S4572761/Nb-*p*-coumaroyltryptamine).

## Data Availability

All data generated or analysed during this study are included in this published article and its Additional files.

## Abbreviations

PTB: Preterm birth
PTL: Preterm labor
IUI: Intrauterine inflammation
LPS: Lipopolysaccharide
OTU: Operational taxonomic unit
VIP: Variable importance in projection
PCA: Principal component analysis
OPLS-DA: Orthogonal projections to latent structures discriminant analysis
NMDS: Nonmetric multidimensional scaling analysis
POS: Positive
NEG: Negative
