# Supplementary figures and images for: Potential roles of the interactions between gut microbiota and metabolites in LPS-induced intrauterine inflammation (IUI) and associated preterm birth (PTB)
Source: J Transl Med. 2024 Jan 2;22:7. doi: 10.1186/s12967-023-04603-8 (PMC10762855; doi:10.1186/s12967-023-04603-8)

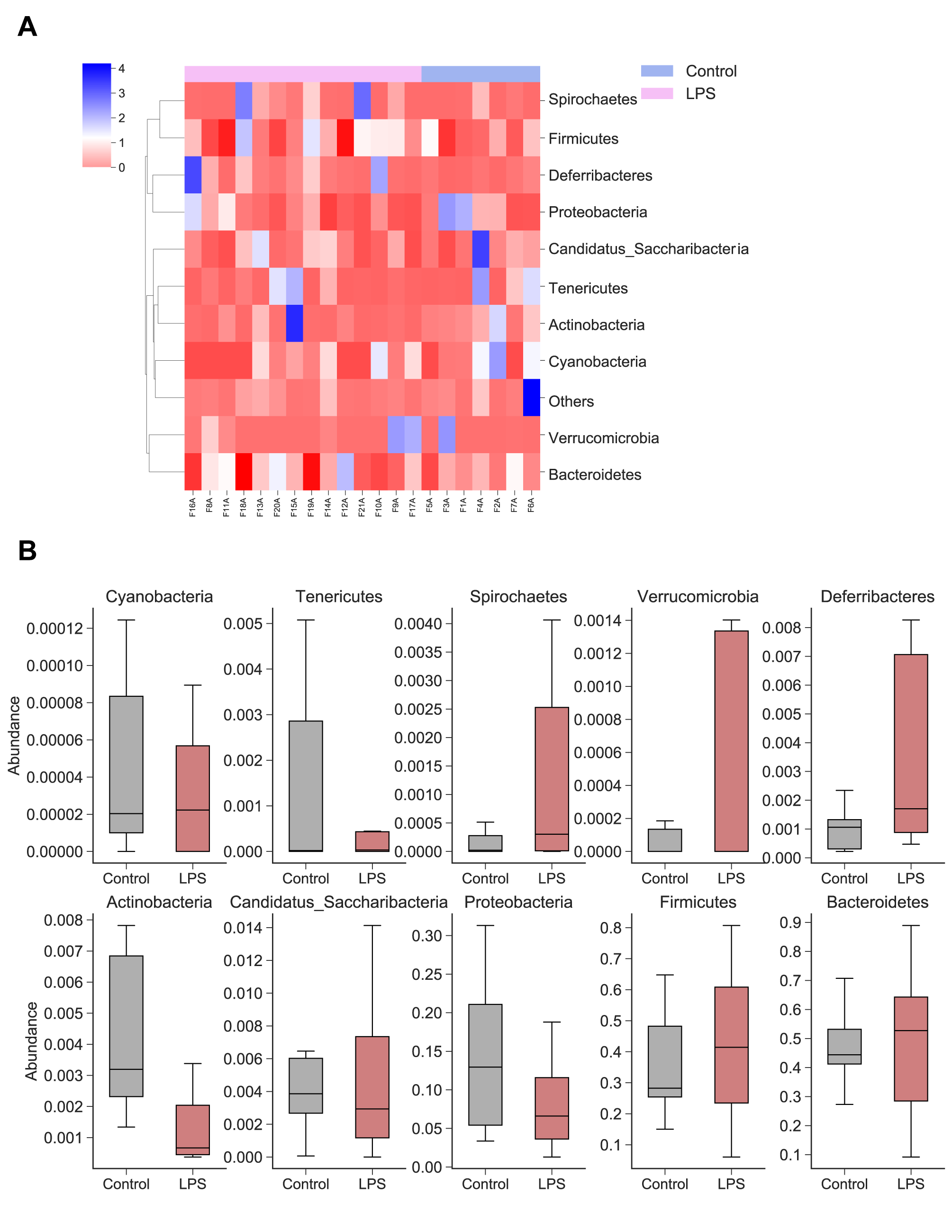

Supplement: Supplementary file 1 — Additional file 1: Figure S1. Annotation on intestinal microbiota at the phylum level. (A) Phyla of intestinal microbiota in mice from different groups. (B) Relative abundance of these phyla of intestinal microbiota. [file 12967_2023_4603_MOESM1_ESM.tif]

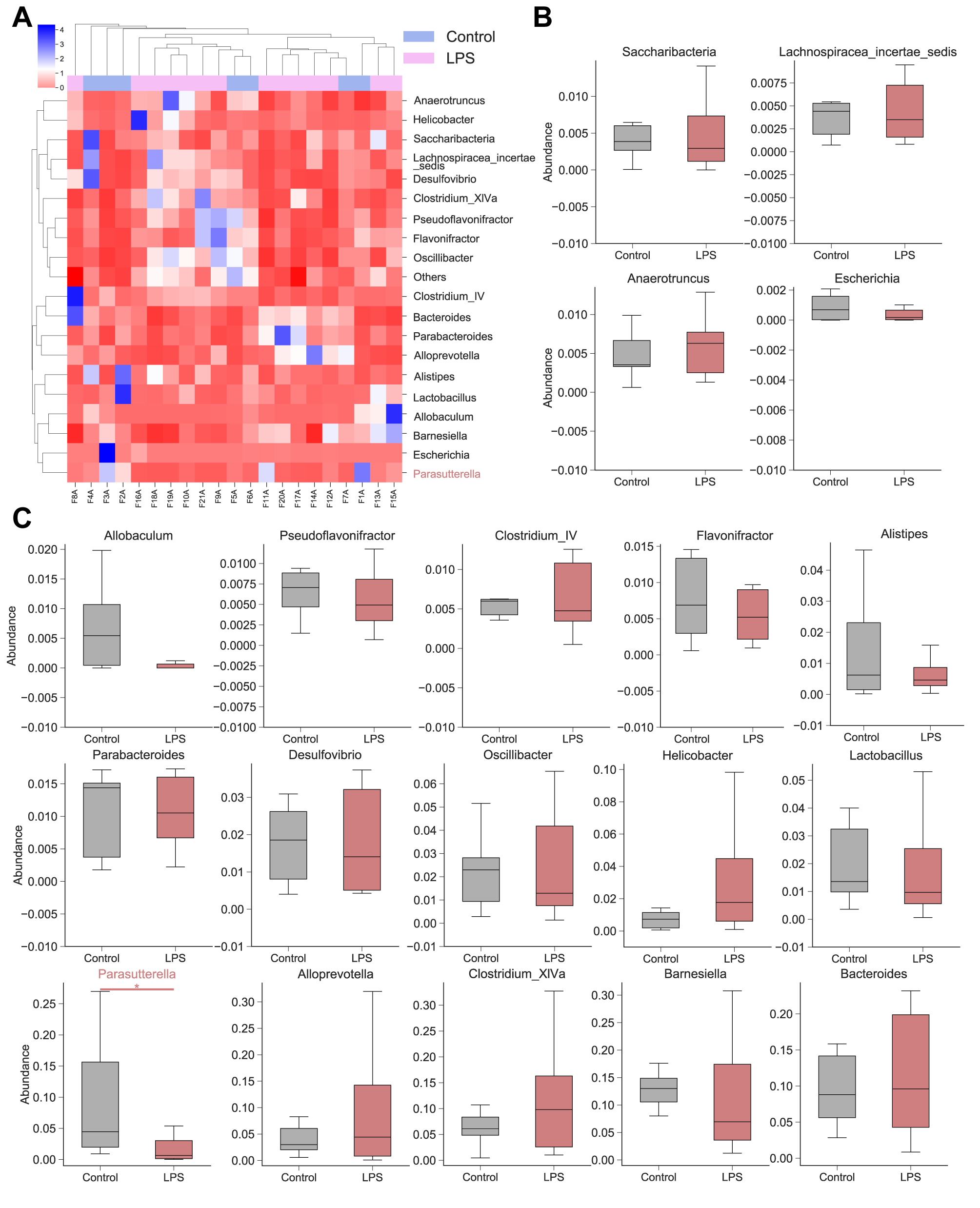

Supplement: Supplementary file 2 — Additional file 2: Figure S2. Annotation on intestinal microbiota at the genus level. (A) Genera of intestinal microbiota in mice from different groups. (B, C) Relative abundance of these genera of intestinal microbiota. *p < 0.05. [file 12967_2023_4603_MOESM2_ESM.tif]

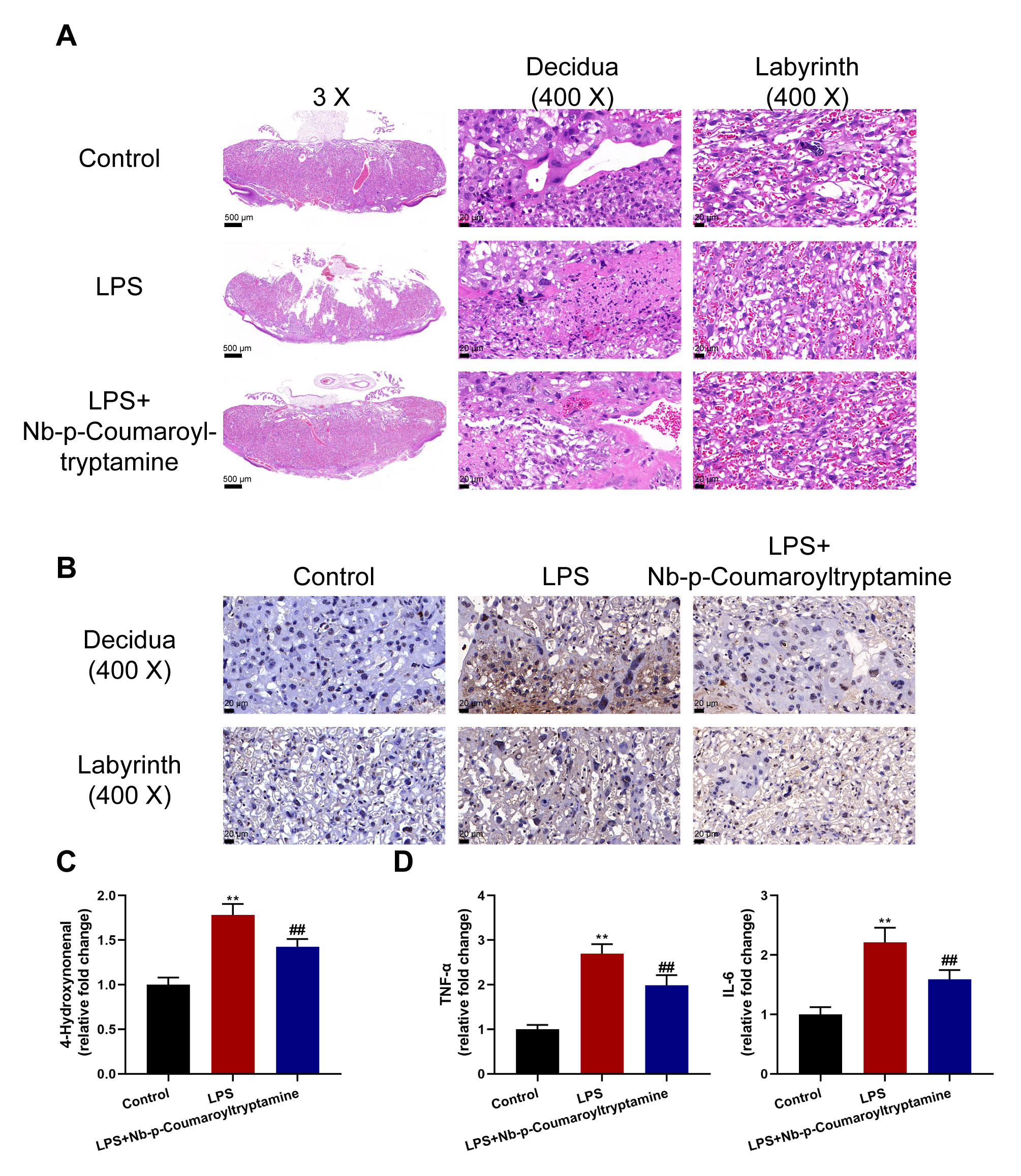

Supplement: Supplementary file 3 — Additional file 3: Figure S3. Effects of Nb-p-coumaroyltryptamine (s4572761) on intrauterine inflammatory phenotypes affecting perinatal adverse reactions. Animal models were established and Nb-p-coumaroyltryptamine treatment was administrated as described in the M&M section. (A) Histopathological characteristics of mice placenta tissues were examined using H&E staining; decidua and labyrinth zones are shown. (B) Apoptosis in the decidua and labyrinth zones of mouse placenta tissues was examined using TUNEL staining. (C) Placental oxidative stress marker 4-hydroxynonenal levels in mice placenta tissues were examined using ELISA. (D) The levels of TNF-α and IL-6 in mice placenta tissues were examined using ELISA. Control = 7, LPS = 14, LPS+ Nb-p-coumaroyltryptamine = 14. **p < 0.01 vs. control group; ##p < 0.01 vs. LPS group. [file 12967_2023_4603_MOESM3_ESM.tif]
