# Supplementary material for: Potential roles of the interactions between gut microbiota and metabolites in LPS-induced intrauterine inflammation (IUI) and associated preterm birth (PTB)
Source: J Transl Med. 2024 Jan 2;22:7. doi: 10.1186/s12967-023-04603-8 (PMC10762855; doi:10.1186/s12967-023-04603-8)
Supplement: Supplementary file 4 — Additional file 4: Table S1. Preterm rate after metabolites intervention (S4572761/Nb-p-coumaroyltryptamine). [file 12967_2023_4603_MOESM4_ESM.docx]

**Table S1 Preterm rate after metabolites intervention (S4572761/Nb-p-Coumaroyltryptamine)**

|  | Control | LPS | LPS + Nb-p-Coumaroyltryptamine | P-value |
| --- | --- | --- | --- | --- |
| Preterm delivery n/N (%) | 0/7 (0%) | 8/14 (57.14%) | 6/14 (42.85%) | 0.040 |
